# Measuring the magnetic topological spin structure of light using an anapole probe

**DOI:** 10.1038/s41377-022-00970-x

**Published:** 2022-10-06

**Authors:** Fanfei Meng, Aiping Yang, Kang Du, Fengyang Jia, Xinrui Lei, Ting Mei, Luping Du, Xiaocong Yuan

**Affiliations:** 1 Nanophotonics Research Centre, Institute of Microscale Optoelectronics, Shenzhen University, 518060 Shenzhen, China; 2 MOE Key Laboratory of Material Physics and Chemistry under Extraordinary Conditions and Shaanxi Key Laboratory of Optical Information Technology, School of Physical Science and Technology, Northwestern Polytechnical University, 710129 Xi’an, China

**Keywords:** Imaging and sensing, Micro-optics

## Abstract

Topological spin structures of light, including the Skyrmion, Meron, and bi-Meron, are intriguing optical phenomena that arise from spin–orbit coupling. They have promising potential applications in nano-metrology, data storage, super-resolved imaging and chiral detection. Aside from the electric part of optical spin, of equal importance is the magnetic part, particularly the H-type electromagnetic modes for which the spin topological properties of the field are dominated by the magnetic field. However, their observation and measurement remains absent and faces difficult challenges. Here, we design a unique type of anapole probe to measure specifically the photonic spin structures dominated by magnetic fields. The probe is composed of an Ag-core and Si-shell nanosphere, which manifests as a pure magnetic dipole with no electric response. The effectiveness of the method was validated by characterizing the magnetic field distributions of various focused vector beams. It was subsequently employed to measure the magnetic topological spin structures, including individual Skyrmions and Meron/Skyrmion lattices for the first time. The proposed method may be a powerful tool to characterize the magnetic properties of optical spin and valuable in advancing spin photonics.

## Introduction

Topological nontrivial spin textures are intriguing in various physical systems, ranging from high energy to condensed matter physics, because they are insusceptible to disturbances that may be exploited for exotic technologies^1–5^. For instance, the magnetic Skyrmions formed by a swirling magnetization in magnetic materials have potential applications in high density magnetic information storage and transfer derived from their excellent stability established through topological protection and by low driven currents^6–10^. Moreover, photonic analogies of magnetic Skyrmions were proposed and demonstrated recently either in 2D form in evanescent waves including steady photonic spin Skyrmions/merons formed through spin–orbit coupling^11–16^ and time-varying field Skyrmion lattice^17,18^ in the absence of optical spin, or in 3D form in propagating structured lights^19^. The deep-subwavelength features of spin structures provide novel tools for optical metrology including high-precision displacement sensing and monitoring of magnetic domains^20^.

The previous Skyrmion structures of light were observed in surface plasmon polaritons, which are transverse magnetic (TM) modes (E-type waves) sustained at a metal-dielectric interface, with electric fields dominating their wave properties. To map the electric-field distributions at near field, many approaches have been proposed in the past including fluorescence imaging^21^, photoemission electron microscopy^14–16,18,22,23^ and near-field scanning optical microscopy (NSOM) with fiber probes or nanoscatters^24–30^. The last was further adapted so that optical spin angular momentum (SAM) associated with the electric fields were able to be measured^31–33^. In addition to the electric part in optical spin, the magnetic part is important as well^34,35^, in particular, for transverse electric (TE) modes (H-type waves), for which magnetic fields mainly determine the optical spin topological properties. Although several near-field mapping techniques have been developed for characterizing the magnetic fields, either using a NSOM probe with specific apex^36–38^ or high refractive index nanoparticles^35,39,40^, they succumb to inevitable influences from the electric field^41,42^. This would affect the vector properties of the measured magnetic field and hence impair the robustness of the system in characterizing the spin topological properties associated with the magnetic fields. The anapole mode of nanoparticles with pure magnetic field response might be a great solution. It has drawn much attention in researches related to near-field optics, nano-optics, etc. because of its intriguing properties, including non-radiation properties realized in dielectric/plasmonic systems^43–46^ and nonlinear responses^47,48^.

**Fig. 1:**
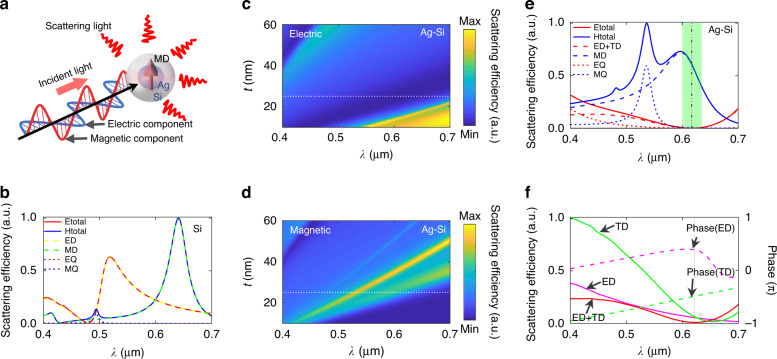
Design of an anapole probe exhibiting a pure magnetic response. **a** Schematic of the Ag core-Si shell nanosphere displaying a pure magnetic dipole with no electric response, **b** typical spectrum of a pure Si-nanosphere of size 160 nm, for the purpose of comparison—the spectral overlap between the electric dipole and magnetic dipole modes hinders the realization of a pure magnetic dipole scatter, **c**, **d** electric and magnetic scattering spectra of the core-shell nanosphere when varying the thickness (*t*) of the outer Si shell (its inner Ag core is fixed at 100 nm), **e** spectra of an optimized nanoparticle under incident light of wavelength 633 nm—the thickness of the outer Si shell equals 25 nm (marked as white dotted lines in **c**, **d**), and **f** spectra of the decomposed electric dipole and toroidal dipole modes including both the magnitude and phase information. The anapole mode at ~617 nm arises through the destructive interference of the electric dipole and toroidal dipole modes. ED electric dipole; MD magnetic dipole; EQ electric quadrupole; MQ magnetic quadrupole; TD toroidal dipole

In this paper, we propose a unique magnetic probe with an anapole mode (hereafter named the anapole probe) that is useful in measuring the topological spin structures of evanescent waves governed by magnetic fields. The probe is composed of an Ag-core and Si-shell nanosphere, for which the excited electric dipole and toroidal dipole modes experience a destructive interference, thereby forming the anapole mode and suppressing scattered radiation caused by electric fields [Fig. 1a]. This anapole mode overlaps with a strong magnetic dipole resonance, which guarantees a high detection efficiency of the magnetic field. A home-built near-field scanning system utilizing the anapole probe was assembled and with which the magnetic topological spin structures of the TE mode were characterized for the first time, including individual photonic Skyrmions and Skyrmion/Meron lattices. The proposed method with high sensitivity and precision may become a valuable tool for studying the underlying physical processes related to the magnetic field components of light, and facilitate the development of applications, including data storage, metrology, optical tweezers, and chiral nanoscopy.

## Results

### Design of the anapole probe

To design an ideal magnetic probe with a strong magnetic field response but no electric response, one has to manipulate the interplay between multipolar modes. Traditionally, for a pure high refractive index nanoparticle such as a Si-nanoparticle, the spectrum overlap between the electric dipole and magnetic dipole modes would hinder the realization of a pure magnetic dipole scatter^42,49^ (see Fig. 1b for a typical resonance spectrum of a Si-nanosphere of size 160 nm). Recently, it was demonstrated that the destructive interference between the electric dipole and toroidal dipole modes inside a Si-nanoparticle, known as an anapole mode, could suppress the electric dipole scattering, which may act as a magnetic scatter in the far field^43,50,51^ [Fig. 1a]. However, the toroidal dipole mode arises when the size of the Si-nanoparticle increases. This would red-shift the magnetic dipole mode, making it difficult to overlap the anapole mode with the magnetic dipole mode in the visible wavelength range^49,52^. In view of this, we considered a core-shell nanoparticle including an Ag-core inside a Si-shell [Fig. 1a]. This design can shift the resonance frequency of the anapole mode to overlap with the magnetic dipole mode in the desired wavelength range. To achieve this ideal anapole probe, the geometric parameters of the nanoparticle were optimized [see Fig. 1c–f)].

Figure 1c, d show the electric and magnetic scattering spectra, respectively, from an Ag-core of fixed radius 100 nm in the visible spectrum versus the thickness of the outer Si-shell. The wide dark ribbon indicates the wide range of the anapole mode [Fig. 1c], which overlaps with the magnetic dipole resonance [Fig. 1d]. Extracting the white dotted lines in Fig. 1c, d where the thickness of the Si-shell is 25 nm, the electric and magnetic scattering spectra are plotted with the decomposed multipoles (up to quadrupoles), as illustrated in Fig. 1e. One sees that the total electric scattering (red line) is suppressed almost to zero over a wide range in wavelengths (highlighted in light green), for which the sum of the electric dipole and toroidal dipole (red dashed line) vanishes and the electric quadrupole scattering (red short dash line) also becomes negligible. To understand the physics underlying the suppression of the electric scattering, different components including the electric dipole and toroidal dipole modes were extracted (see Methods for the calculation) and their magnitudes and phases plotted [Fig. 1f]. One sees clear destructive interference between the two modes within the wavelength region ~617 nm, for which their magnitudes are comparable while their oscillations are out-of-phase. More importantly, the strong magnetic dipole mode [blue dashed line in Fig. 1e] dominates in the anapole mode region, with negligible influence from higher order modes (magnetic quadrupole, blue short dashed line). This situation is significantly different to that for traditional commonly used Si-probes, in which the magnetic dipole mode accompanies a non-negligible electric-field response [Fig. 1b].

### Mapping system for measuring spin-skyrmion textures

**Fig. 2:**
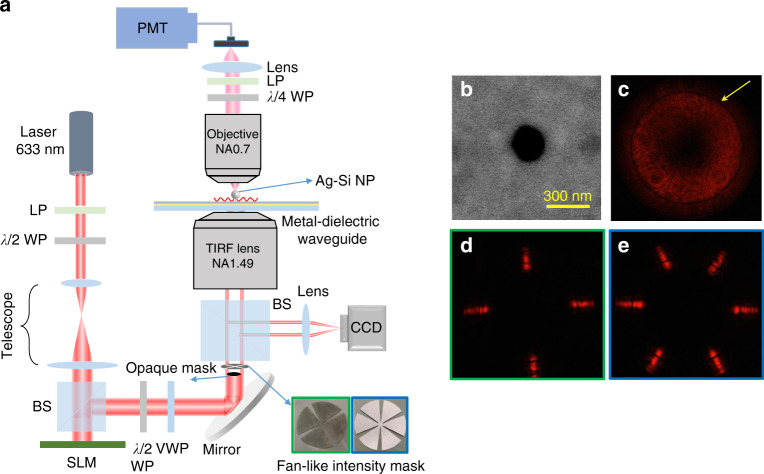
Experimental configuration for validating the effectiveness of the proposed anapole probe and measuring the magnetic photonic spin topological structures. **a** Schematic of the setup, **b** SEM image of the designed core-shell nanoparticle, **c** image of the reflected beam captured at the CCD mounted on the back focal plane of the excitation TIRF objective lens—the dark ring indicates the excitation of a TE mode in the metal-dielectric waveguide by the incident azimuthal polarized beam, and **d**, **e** images of the reflected beams when a fan-like intensity mask with 4-fold symmetry (for generating a spin-Meron lattice) and 6-fold symmetry (for generating a spin-Skyrmion lattice) were inserted into the incident beam. LP linear polarizer; *λ*/2 WP half-wave plate; VWP vortex wave plate; BS beam splitter; SLM spatial light modulator; *λ*/4 WP quarter-wave plate; PMT photo-multiplier tube; CCD charge-coupled device

To validate the effectiveness of the proposed method, the designed magnetic probe was employed to measure the near-field distributions of waveguide modes excited by various tightly focused beams. The near-field scanning system assembled in-house [Fig. 2a] included a linearly polarized He-Ne laser operating at 633-nm-wavelength as the excitation source. The beam was expanded by a telescope structure and illuminated onto a spatial light modulator (SLM) to perform a phase modulation. A half-wave plate was used before the SLM to provide beam polarization matching with the SLM. A pair of half-wave plate and a vortex wave plate was then inserted into the beam to change the beam’s linear polarization to azimuthal polarization. The modulated beam was finally tightly focused onto a metal-dielectric waveguide structure using a total-internal reflection (TIRF) objective lens (Olympus, 100×, NA = 1.49).

**Fig. 3:**
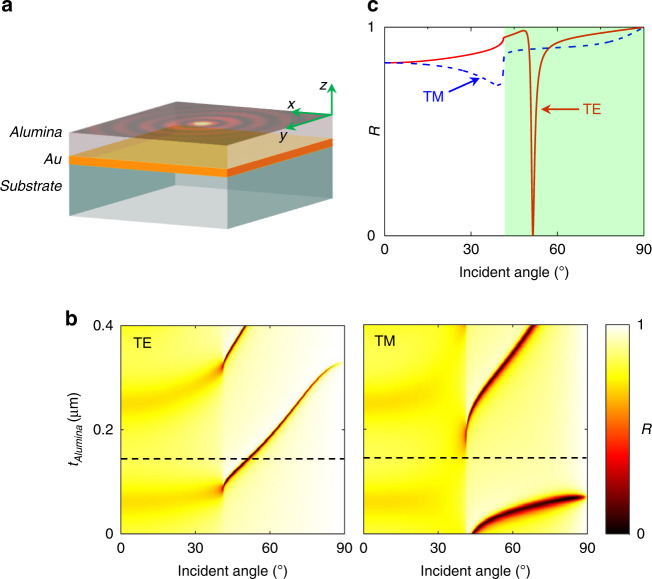
Designed metal-dielectric waveguide. **a** Schematic showing a thin gold film sandwiched by an alumina layer and a silica substrate, **b** contour maps of the reflectivity (*R*) of the metal-dielectric waveguide structure calculated using transfer matrix theory^57,58^ for both the TE and TM modes, **c** reflectivity curves when the thickness of alumina layer (*t*_Alumina_) equals 145 nm (corresponding to the black dashed lines in **b**). Only the TE mode exists in the metal-dielectric waveguide. The thickness of the gold film was fixed at 40 nm in the calculation

The metal-dielectric waveguide structure is composed of a thin layer of Au film of thickness 40 nm, sandwiched below by a silica substrate and above by an alumina layer of thickness 145 nm [Fig. 3a]. This structure supports only the TE mode [Fig. 3b, c], with mode properties dominated by the magnetic field. To verify the excitation of the waveguide mode, a charge-coupled-device (CCD) camera was mounted at the back focal plane of the TIRF objective lens to capture the reflected beam from the metal-dielectric waveguide structure. The dark ring at the reflected beam cross-section [Fig. 2c] registers the excitation of the TE mode in the metal-dielectric waveguide structure by the incident azimuthal polarized beam. The designed core-shell nanoparticle, with an Ag-core radius of 100 nm and an outer Si-shell thickness of 25 nm, was immobilized on the surface of the alumina layer. A scanning electron microscope (SEM) image of the nanoparticle is shown in Fig. 2b. The nanoparticle was employed as a near-field probe to scatter the evanescent waves at the alumina surface into the far-field for detection. The scattered light from the nanoparticle was collected by another objective lens (Olympus, 60×, NA = 0.7), and then directed to a photo-multiplier tube (Hamamatsu R12829) over a fiber coupler for signal analysis. The near-field distributions of the waveguide mode were finally obtained by raster scanning the Piezo stage (Physik Instrumente, P-545) on which the metal-dielectric waveguide sample was mounted.

### Mapping the in-plane magnetic near fields

**Fig. 4:**
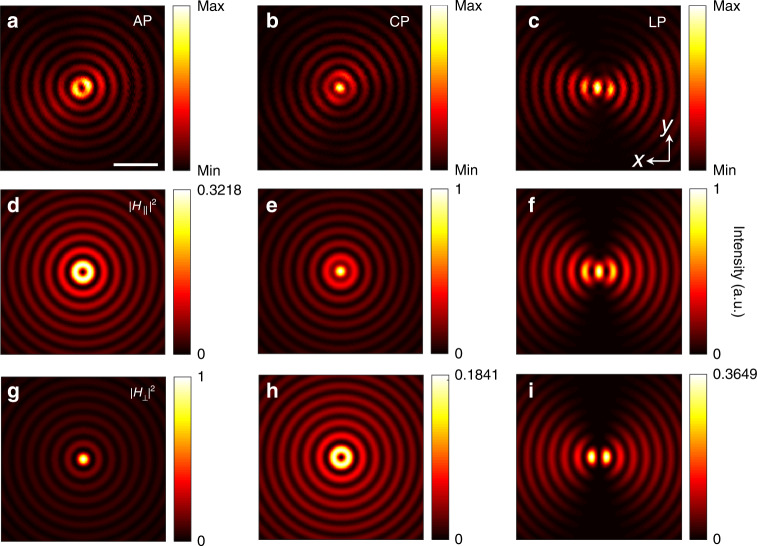
Mapping the in-plane magnetic near fields. **a**–**c** mapped intensity distributions of various TE-type waveguide modes excited by the different incident polarized beams—from left to right, azimuthally polarized (AP), circularly polarized (CP), and linearly polarized (LP) beams; **d**–**i** calculated in-plane (**d**–**f**) and out-of-plane (**g**–**i**) magnetic field component distributions. The experimental results indicate the in-plane magnetic field sensitivity of the anapole probe. The scalar bar in **a** is 1 μm; all images have the same area

Figure 4a shows the mapped intensity pattern of the waveguide mode excited by an azimuthal polarized beam. One can see a cylindrically symmetric standing wave with a series of concentric rings. This was formed due to the interference of TE waves excited from all of the azimuthal directions by the azimuthal polarized beam. Note that the intensity vanishes at the geometric center. This indicates that the mapped field should be the transversal (in-plane) magnetic field component of the waveguide mode, and the null-intensity at the center arises from the polarization singularity. To verify this, the transversal and longitudinal magnetic field components were calculated theoretically using the Richard–Wolf vectorial diffraction theory^53^. The results are shown in Fig. 4d, g, respectively. The mapped intensity pattern accords well with the calculated transversal magnetic field. This transversal-field sensitivity of the probe occurs because of the radiative property of the magnetic dipole above the film. A horizontal dipole radiates at small angles with respect to the normal direction, whereas a vertical dipole radiates at relatively larger angles^11,42,54^. Therefore, by employing a collection objective, which acts as a low-pass filter, the radiation from the horizontal dipole excited by the in-plane magnetic field is well-extracted. To confirm the in-plane field sensitivity of the probe, the incident beam was converted to circular polarization by replacing the vortex wave plate with a quarter-wave plate, and to linear polarization by removing the vortex wave plate. The mapped intensity patterns for both are shown in Fig. 4b, c, respectively; the corresponding calculated magnetic field components are shown in Fig. 4e, f for the transversal components and in Fig. 4h, i for the longitudinal components. The experimental results verify again the in-plane magnetic field sensitivity of the probe and the outstanding signal-to-noise ratio of the technique resulting from the excitation of anapole mode.

### Measuring photonic spin topological textures of TE-type evanescent waves

With the ability to measure in-plane magnetic fields, the spin properties of the TE waveguide mode can now be characterized in a similar manner as the TM-type surface plasmon mode^11^. Supposing that the waveguide mode is propagating in the *xy*-plane and exponentially decaying in the upper half-plane *z* > 0 [Fig. 3a], the electromagnetic field components then satisfy

1 $$\begin{array}{*{20}{c}} {E_x = \frac{{i\omega \mu }}{{\beta ^2}}\frac{{\partial H_z}}{{\partial y}},} & {E_y = - \frac{{i\omega \mu }}{{\beta ^2}}\frac{{\partial H_z}}{{\partial x}}} \\ {H_x = - \frac{{k_z}}{{\beta ^2}}\frac{{\partial H_z}}{{\partial x}},} & {H_y = - \frac{{k_z}}{{\beta ^2}}\frac{{\partial H_z}}{{\partial y}}} \end{array}$$

where *β, k*_*z*_, and *k* denote real numbers associated with the in-plane, out-of-plane, and total wave-vector, respectively, satisfying condition *β*^2^ = *k*^2^ + *k*_*z*_^2^, *ω* denotes the angular frequency of the electromagnetic field, and *μ* the permeability of the propagating medium. For a TE-type evanescent wave, the wave properties are determined by the out-of-plane magnetic field component *H*_*z*_, which satisfies the Helmholtz equation, ∇^2^*H*_*z*_ + *k*^2^*H*_*z*_ = 0. Moreover, the out-of-plane electric field *E*_*z*_ vanishes, and the in-plane electric and magnetic field obey the relations,

2 $$E_x = - \frac{{i\omega \mu }}{{k_z}}H_y,E_y = \frac{{i\omega \mu }}{{k_z}}H_x$$

With the above electromagnetic field components, the SAM of the wave can be calculated using${{{\mathbf{S}}}} = \left\{ {\varepsilon {{{\mathbf{E}}}}^ \ast \times {{{\mathbf{E}}}} + \mu {{{\mathbf{H}}}}^ \ast \times {{{\mathbf{H}}}}} \right\}/4\omega i$, where * denotes complex conjugation and *ε* denotes the permittivity of the medium. As a result, the *z*-component of the SAM for a TE mode is related to the in-plane magnetic field components by

3 $$S_z = S_z^E + S_z^H = \frac{\mu }{{4\omega i}}\frac{{\beta ^2}}{{k_z^2}}\left( {H_x^ \ast \,H_y - H_y^ \ast \,H_x} \right) = \frac{\mu }{{4\omega }}\frac{{\beta ^2}}{{k_z^2}}\left( {I_{{{{\mathrm{RCP}}}}} - I_{{{{\mathrm{LCP}}}}}} \right)$$

where *I*_RCP_ and *I*_LCP_ denote the right-handed and left-handed circular polarized components of the in-plane magnetic field of the waveguide mode.

**Fig. 5:**
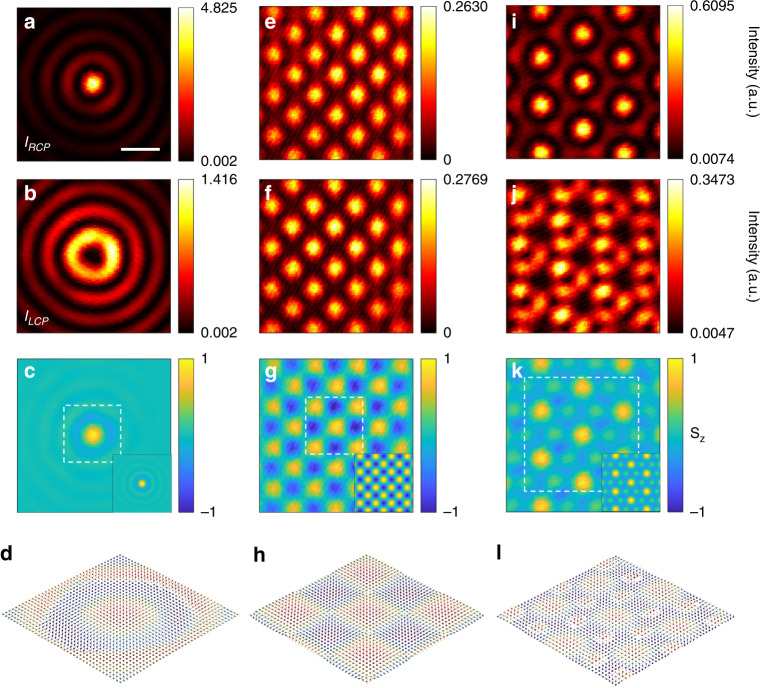
Measurement of the various photonic spin topological structures of the TE mode dominated by magnetic fields. **a**–**d** individual spin-Skyrmion, **e**–**h** spin-Meron lattice, **i**–**l** spin-Skyrmion lattice. From top to bottom are: the RCP components of the in-plane magnetic fields (*I*_RCP_), the LCP component (*I*_LCP_), the normalized *z*-component SAM (*S*_*z*_), and the reconstructed spin vector patterns. Insets in **c**, **g** and **k** are calculated *S*_*z*_ distributions for comparison. The scalar bar in **a** is 0.5 μm and all of contour maps have the same area. Regions of **d**, **h** and **l** correspond to the white dashed boxes in **c**, **g** and **k**, respectively

We then use the above experimental setup to measure various topological spin structures of TE modes dominated by the magnetic field. In the excitation section of the setup, a spiral phase with topological charge of 1 was coded onto the SLM to convert the azimuthal polarized beam to an azimuthal polarized vortex beam. The excited waveguide mode in this circumstance generates a Bessel vortex beam where the spin–orbit coupling results in the formation of a TE-type photonic Skyrmion. To characterize this new type of spin Skyrmion, a quarter-wave plate (*λ*/4) and a linear polarizer were inserted in the collection section of the setup [Fig. 2a]. By controlling the angle formed by the axes of the *λ*/4 wave-plate and the linear polarizer plate to alternate between ±45° using a high-speed motorized rotation mount (Thorlabs, DDR25), the intensity of the right-handed (*I*_RCP_) and left-handed (*I*_LCP_) circular polarized components of the in-plane magnetic fields can be extracted. Figure 5a, b show the measured *I*_RCP_ and *I*_LCP_ of the excited waveguide mode under the illumination with the azimuthal polarized vortex beam. The normalized *z*-component SAM (*S*_*z*_) distribution can then be obtained from Eq. (3); the result is shown in Fig. 5c. For the purpose of comparison, the theoretical *S*_*z*_ distribution was calculated using the Richard–Wolf theory^53^. We found an excellent match between the experimental and theoretical results [Fig. 5c, inset]. Furthermore, the in-plane SAM components (*S*_*x*_ and *S*_*y*_) can be reconstructed from the measured *S*_*z*_ component using the same methodology demonstrated in refs. ^12,32^. The spin vector pattern was then obtained [Fig. 5d] in which an individual TE-type photonic spin Skyrmion is visible and analyzed using the mapping technique developed for the purpose.

Finally, to form and analyze the spin-Meron and spin-Skyrmion lattices of the TE mode, fan-like intensity masks with different symmetries were inserted into the azimuthal polarized vortex beam [Fig. 2a; green and blue insets]. Back focal plane images [Fig. 2d, e] were obtained of the reflected beam under the illumination of the azimuthal polarized vortex beam after using these intensity masks. Following the same procedure, a square spin-Meron lattice with 4-fold symmetry [Fig. 5g] and a hexagonal spin-Skyrmion lattice with 6-fold symmetry [Fig. 5k] were characterized and reconstructed spin vector distributions developed [Fig. 5h, l]; they correspond to the dashed boxes in Fig. 5g, k, respectively. For the Meron lattice, the spin state is orientated either downward or upward in the center of each unit cell but is horizontal at the edges (Skyrmion number *n* = ±1/2). With a 6-fold symmetry, two sets of hexagonal sub-lattices for the Skyrmion were obtained with the spin states gradually changing from an upward state in the center to a downward state at the edge (Skyrmion number *n* = 1). All the measured patterns were reproduced in theoretical calculations with high reliability and predictability.

## Discussion

We designed an anapole probe to measure the magnetic part of various photonic spin topological textures. The probe is a mixed nanosphere composed of an Ag-core and outer Si-shell, for which the magnetic dipole resonance dominates completely at the desired wavelength along with the elimination of electric-field scattering by the anapole mode. A designed nano-probe was incorporated into our home-built near-field mapping system to map with high robustness the in-plane magnetic field distributions of various tightly focused waveguide modes. Furthermore, photonic spin topological textures formed by the TE-type evanescent waves, including individual Skyrmion, and Skyrmion/Meron lattices with different symmetries, were measured for the first time. The proposed method will facilitate the study of physical processes related to the magnetic components of electromagnetic fields and be valuable in advancing spin photonics.

## Materials and methods

### Numerical simulations

To perform multipole decomposition of the scattering spectra for the Ag-core and Si-shell nanospheres, 3D finite-difference time-domain simulations were conducted using commercial software Lumerical. The optical constants for the Ag-core and Si-shell were taken from published data^55^. Perfectly matched layers were used to simulate open space, and the core-shell nanosphere was illuminated by a normal-incident plane wave polarized along the *x*-axis. The center of the nanosphere was set as the origin for the coordinate system. The electric fields **E**(***r***) at every discretized point ***r*** inside the core-shell nanosphere were recorded using a 3D frequency-domain field monitor. By introducing the polarization current **J**(***r***), the electric, toroidal, and magnetic multipole moments (**P**, **T**, and **m**, respectively) may be calculated from^56^:

4 $$\begin{array}{l}{{{\mathbf{J}}}}({{{\boldsymbol{r}}}}) = - i\omega \varepsilon _0[\varepsilon _r({{{\boldsymbol{r}}}}) - \varepsilon _d]{{{\bf{E}}}}({{{\boldsymbol{r}}}})\\\quad\,{{{\mathbf{P}}}}\;{{{\mathrm{ = }}}}\;\frac{1}{{i\omega }}{\int}_V {{{\mathbf{J}}}} {\mathrm{d}}V\\\quad\,{{{\mathbf{T}}}} = \frac{1}{{10c}}{\int}_V {[({{{\mathbf{r}}}} \cdot {{{\mathbf{J}}}}){{{\mathbf{r}}}} - 2r^2{{{\mathbf{J}}}}]{\mathrm{d}}V} \\\quad {{{\mathbf{m}}}} = \frac{1}{{2c}}{\int}_V {({{{\mathbf{r}}}} \times {{{\mathbf{J}}}})} {\mathrm{d}}V\\\;\; Q_{ij}^e = \frac{1}{{2i\omega }}{\int}_V {[r_iJ_j} + r_jJ_i - \frac{2}{3}\delta _{ij}({{{\mathbf{r}}}} \cdot {{{\mathbf{J}}}})]{\mathrm{d}}V\\\;\; Q_{ij}^m = \frac{1}{{3c}}{\int}_V {[({{{\mathbf{r}}}} \times {{{\mathbf{J}}}})_i{{{\mathbf{r}}}}_j} + ({{{\mathbf{r}}}} \times {{{\mathbf{J}}}})_j{{{\mathbf{r}}}}_i]{\mathrm{d}}V\end{array}$$

where *ε*_*r*_(***r***) and *ε*_*d*_ denote the relative permittivities of the nanosphere and air background, respectively, *ε*_0_ denotes the vacuum permittivity, *ω* the angular frequency, $Q_{ij}^e$ and $Q_{ij}^m$ denote the electric and magnetic quadrupole moment components with *i*, *j* = *x, y, z*. The total scattering cross-section *σ*_sca_ and the total scattering efficiency *Q*_sca_ are calculated from

5 $$\begin{array}{l}\sigma _{{\mathrm{sca}}} \simeq \frac{{k_0^4}}{{6\pi \varepsilon _0^2\left| {{{{\mathbf{E}}}}_0} \right|^2}}\left| {{{{\mathbf{P}}}} + \frac{{ik_0\varepsilon _d}}{c}{{{\mathbf{T}}}}} \right|^2 + \frac{{k_0^4\varepsilon _d\mu _0}}{{6\pi \varepsilon _0\left| {{{{\mathbf{E}}}}_0} \right|^2}}\left| {{{\mathbf{m}}}} \right|^2{{{\mathrm{ + }}}}\frac{{k_0^6\varepsilon _d}}{{720\pi \varepsilon _0^2\left| {{{{\mathbf{E}}}}_0} \right|^2}}\mathop {\sum}\limits_{ij} {\left| {Q_{ij}^e} \right|} ^2 + \frac{{k_0^6\varepsilon _d^2\mu _0}}{{80\pi \varepsilon _0\left| {{{{\mathbf{E}}}}_0} \right|^2}}\mathop {\sum}\limits_{ij} {\left| {Q_{ij}^m} \right|} ^2\\ Q_{{\mathrm{sca}}} = \frac{{\sigma _{{\mathrm{sca}}}}}{{\pi a^2}}\end{array}$$

where **E**_0_ denotes the incident electric field and *a* the outer radius of the nanosphere.

The scattering efficiency curves (Fig. 1) were all normalized for ease of comparison. The scattering efficiencies and phases for the electric dipole and toroidal dipole [Fig. 1f] were calculated using

6 $$\begin{array}{l}\sigma _{{\mathrm{sca}}}^{ED} = \frac{{k_0^4}}{{6\pi \varepsilon _0^2\left| {{{{\mathbf{E}}}}_0} \right|^2}}\left| {{{\mathbf{P}}}} \right|^2,\sigma _{{\mathrm{sca}}}^{TD} = \frac{{k_0^4}}{{6\pi \varepsilon _0^2\left| {{{{\mathbf{E}}}}_0} \right|^2}}\left| {\frac{{ik_0\varepsilon _d}}{c}{{{\mathbf{T}}}}} \right|^2\\ {{{\mathrm{Phase(ED)}}}} = \arg (P_x){{{\mathrm{,}}}}\,{{{\mathrm{Phase(TD)}}}} = \arg (\frac{{ik_0\varepsilon _d}}{c}T_x)\end{array}$$

where arg(•) indicates the argument of the complex number.

### Sample preparation

The metal-dielectric waveguide structure was fabricated through electron-beam evaporation. The Au layer (thickness: 40 nm) and the alumina film (thickness: 145 nm) were deposited on a cover slip. The Ag-core and Si-shell nanoparticle was customized by XFNANO Materials Tech Co., Ltd. The solvent was deionized-water, and ultrasonic oscillation for ~30 min was needed before use. After ~10^4^ times of dilution, the diluted nanoparticle suspension was dropped on the metal-dielectric waveguide structure and vapored naturally. Then the nanoparticles immobilized steadily on the surface of the metal-dielectric waveguide were left after a rinse using deionized-water. Once dried using nitrogen gas, the samples are ready to use. The sample was checked by a dark-field microscopy, and the proper concentration was that there exists only one nanoparticle in the region of detection.
